# The relationship between higher social trust and lower late HIV diagnosis and mortality differs by race/ethnicity: results from a state-level analysis

**DOI:** 10.7448/IAS.20.01/21442

**Published:** 2017-04-06

**Authors:** Yusuf Ransome, Ashley Batson, Sandro Galea, Ichiro Kawachi, Denis Nash, Kenneth H. Mayer

**Affiliations:** ^a^ Social and Behavioral Sciences, Harvard T.H. Chan School of Public Health, Boston, MA, USA; ^b^ Department Behavioral and Social Sciences, Brown School of Public Health, Providence, RI, USA; ^c^ School of Public Health, Boston University, Boston, MA, USA; ^d^ Institute for Implementation Science in Population Health, City University of New York, New York, NY, USA; ^e^ Graduate School of Public Health and Health Policy, City University of New York, New York, USA; ^f^ The Fenway Institute, Fenway Health, Boston, MA, USA; ^g^ Department of Medicine, Beth Israel Deaconess Medical Center/Harvard Medical School, Boston, MA, USA

**Keywords:** Social capital, late HIV diagnosis, AIDS mortality, race/ethnicity, MSM, key populations, surveillance, HIV care continuum

## Abstract

**Introduction**: Black men who have sex with men (MSM) continue to suffer a disproportionate burden of new HIV diagnoses and mortality. To better understand some of the reasons for these profound disparities, we examined whether the association between social trust and late HIV diagnosis and mortality differed by race/ethnicity, and investigated potential indirect effects of any observed differences.

**Methods**: We performed generalized structural equation modelling to assess main and interaction associations between trust among one’s neighbours in 2009 (i.e. social trust) and race/ethnicity (Black, White, and Hispanic) predicting late HIV diagnosis (a CD4 count ≤200 cell/µL within three months of a new HIV diagnosis) rates and all-cause mortality rates of persons ever diagnosed late with HIV, across 47 American states for the years 2009–2013. We examined potential indirect effects of state-level HIV testing between social trust and late HIV diagnosis. Social trust data were from the Gallup Healthways Survey, HIV data from the Centers for Disease Control and Prevention, and HIV testing from the Behavioral Risk Factor Surveillance System. Covariates included state-level structural, healthcare, and socio-demographic factors including income inequality, healthcare access, and population density. We stratified analysis by transmission group (male-to-male, heterosexual, and injection drug use (IDU)).

**Results**: States with higher levels of social trust had lower late HIV diagnosis rates: Adjusted Rate Ratio [aRR] were consistent across risk groups (0.57; 95%CI 0.53–0.62, male-to-male), (aRR 0.58; 95%CI 0.54–0.62, heterosexual) and (aRR 0.64; 95%CI 0.60–0.69, IDU). Those associations differed by race/ethnicity (all *p* < 0.001). The associations were most protective for Blacks followed by Hispanics, and least protective for Whites. HIV testing mediated between 18 and 32% of the association between social trust and late HIV diagnosis across transmission group but for Blacks relative to Whites only. Social trust was associated with lower all-cause mortality rates and that association varied by race/ethnicity within the male-to-male and IDU transmission groups only.

**Conclusions**: Social trust may promote timely HIV testing, which can facilitate earlier HIV diagnosis, thus it can be a useful determinant to monitor the relationship with HIV care continuum outcomes especially for racial/ethnic minority groups disproportionately infected by HIV.

## Introduction

Disparities in HIV/AIDS incidence and prevalence persist by race/ethnicity, transmission group, and geography in the United States (US) [1,2]. Blacks comprise 13% of the US population yet have higher rates of new HIV diagnosis than Whites within the three major transmission groups [3].

Beyond individual factors [4]; structural, social, and psychological factors at the aggregate level drive HIV transmission [5–7]. For instance, income inequality and racial residential segregation negatively impact HIV care continuum by delaying HIV diagnosis [8,9]. Higher levels of structural/psychological factors such as sexual minority stigma and experienced discrimination are also associated with lower HIV prevention behaviours and higher HIV incidence, particularly among key populations [8,10].

While the definition of social cohesion and social capital vary in the literature according to different theoretical underpinnings [11–13], it can be considered a single construct [14] broadly defined as contextual-level (i.e. neighbourhood, state) networks and resource that individuals can draw upon for mutual support [14,15]. Social cohesion and capital (hereafter social cohesion/capital) has been theorized [6] and documented empirically to have protective associations with HIV-related behaviours and HIV care continuum outcomes [16–19] among key populations [20,21].

One indicator of social cohesion/capital is mutual trust among neighbours [11,12], which can facilitate the willingness of individuals to work towards achieving or realize collective goals that activate neighbourhood collective efficacy [22]. Mechanisms linking social cohesion/capital to HIV include increased supportive social norms, higher information exchange, reduced HIV/AIDS stigma resulting in timely HIV testing and engagement in HIV care [20,23–25]. Next, the association between social cohesion/capital and HIV care continuum indicators differs across subgroups, for example sex [16,26,27]. We do not know, however, whether that association differs by race/ethnicity across the major transmission groups in the US.

We examined the association between social trust in relation to late HIV diagnosis and (b) all-cause mortality. Those two outcomes span both ends of the HIV care continuum [28,29], so juxtaposing them can provide a sense whether social trust operates through mechanisms that affects sequential steps [30] even outside the continua [29]. We also examined whether the association between social trust and those indicators differed by race/ethnicity. Third, based on theory [21,31], we examined whether HIV testing mediated the association between social trust and late HIV diagnosis. We then estimated the potential reductions in late HIV diagnosis for social trust in comparison to other selected social and structural predictors.

**Figure 1.**
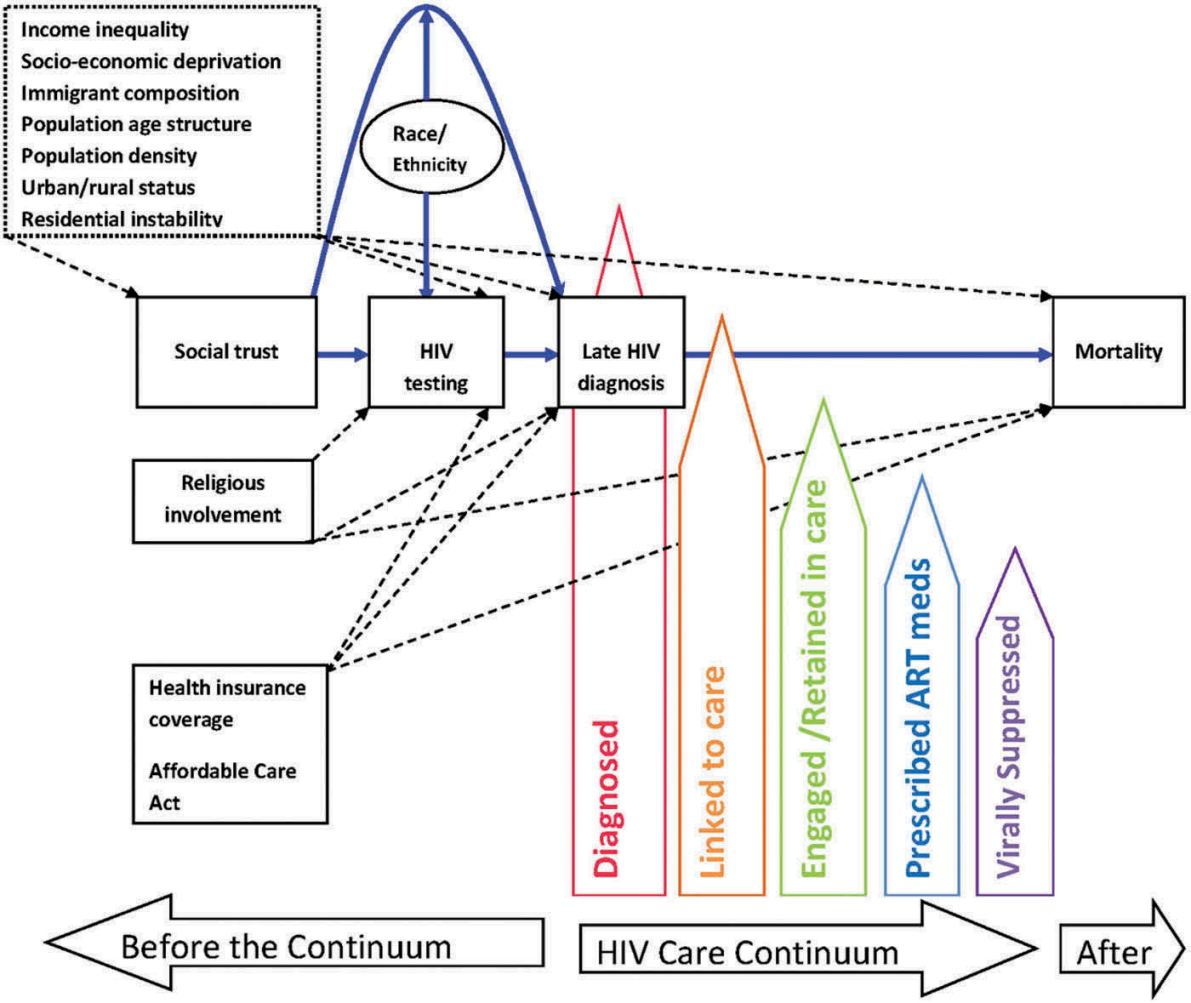
Heuristic model of the relationship among race/ethnicity, social trust, HIV testing, covariates and HIV Care Continuum indicators. Solid paths in blue are those tested in this study. Dotted paths are also those estimated or plausible. Dotted box represents factors theorized as exogenous to social trust, but controlled for in the analysis since all data were cross-sectional. Notes: this model does not represent every possible pathway but those that are related to this study. HIV care continuum is based on AIDS.gov metrics.

American states is the unit of analysis because at this level, economic, legal, and health policies are enacted, which have downstream impacts on communities, social and sexual networks, and individuals [5]. State-level policies also impact HIV testing coverage [32] and have direct impacts on HIV care continuum indicators [33]. Furthermore, states are a well-documented unit of analysis in prior social capital and HIV research [18,34,35]. In Figure 1, we present a heuristic model that guided our analyses.

We theorize that higher social trust among individuals may facilitate higher collective efficacy/action to lobby for legislative policies [36] that fund resources to improve HIV testing coverage and treatment [37] and plausibly also to increase one’s own HIV testing behaviour [38]. Greater coverage could then translate into timely HIV testing and ultimately to lower rates of late HIV diagnosis, earlier enrolment into care, and subsequently lower mortality in the population.

## Methods

### Study population

We accessed HIV surveillance data from the National Center for HIV/AIDS, Viral Hepatitis, STD, and TB Prevention (NCHHSTP) Atlas, which is an interactive online tool that utilizes Centers for Disease Control and Prevention (CDC) surveillance data [39]. The study used years 2009–2013 for 47 states by race/ethnicity (Black, White, and Hispanic) and the three major transmission groups (male-to-male, heterosexual (hereafter MSM), injection drug use (IDU)). We excluded Alaska, Hawaii, and New Hampshire because of missing or suppressed HIV/AIDS or social trust data, and Washington DC because of extremely high outlier values on the HIV/AIDS data. The study population was *N* = 705 (i.e., 47 states over 5 years stratified by 3 race/ethnic groups within each of the 3 transmission groups, except in mortality, *N* = 704 for MSM, and *N* = 693 due to no cases in some groups (see Supplement Table 2 for notes). All data reflect most up-to-date cases diagnosed through 31 December 2014 and reported as of 31 July 2015.

### Measures

#### Late HIV diagnosis rate

We calculated this using the numerator of the count of HIV-infected individuals defined by the CDC as documentation of an AIDS-defining condition or either a CD4 count of <200 cells/μL or a CD4 percentage of total lymphocytes of <14 within 3 months of an initial HIV infection [40]. The denominator was the year-specific total of the general population for each of the 5 years within each of the 47 states. Intercensal population data were retrieved online from the American Community Survey (ACS) 1-year estimates [41]. We calculated rates for each year per 100,000 persons in the population.

#### All-cause mortality rate

All-cause mortality rate for persons with diagnosed HIV infection ever classified as Stage 3 (AIDS). We calculated this measure via a similar process above. The numerator was the total deaths from all causes (i.e. death may not be related to HIV) for individuals ever classified as Stage 3 (AIDS) who have died in a given time period [42]. The denominator is the year-specific number of persons living with HIV/AIDS (PLWHA), which potentially includes individuals ever diagnosed late with HIV. We calculated rates for each year and state per 1000 PLWHA.

#### Social trust

We operationalized this as the state-level aggregate proportion of people who expressed trust in one’s neighbour, when asked: “If you lost a wallet or purse that contained two hundred dollars and it was found by a neighbour, do you think it would be returned with the money in it, or not?” Response option was yes or no. Data were from the 2009 Gallup Healthways Survey retrieved online from a report on Gallup’s website [43]. The survey was based on landline and cell phone interviews with 178,543 adults aged 18 and older from the non-institutionalized population [43]. The social trust variable was based on an empirically supported [44] reliable and valid indicator within Putnam and Coleman’s definition of social capital [11,12] and reflected the trust component of social cohesion within Robert Sampson’s definition of collective efficacy [22].

#### HIV testing

We operationalized this as the state-level aggregate age-adjusted race/ethnic-specific prevalence of recent HIV testing (last 12 months). We derived the measure based on two questions within the Behavioral Risk Factor Surveillance System (BRFSS) Surveys for 2009 to 2011. We based age-adjustment on Census 2010 population estimates. The first question was: “have you been tested for HIV? Do not count tests you may have had as part of a blood donation. Include testing fluid from your mouth.” The second question was: “not including blood donations, in what month and year was your last HIV test?” Responses were among persons aged 18–64 years. We used date of survey interview and the two questions to construct the proportion of persons who reported HIV testing in the past 12 months. BRFSS is a national probability survey yielding 49% response rate for landline users. The survey, conducted among over 400,000 adults, assessed health-related risk behaviours, chronic diseases, and use of preventative services in the U.S. Data are publicly available [45]. We only used those three years because in 2012 the BRFSS began using a new survey methodology and recommended that 2012 estimates should not be combined with estimates produced from prior years [46].

#### Income inequality

Erodes social capital [47]. We operationalized this measure using the GINI coefficient, which ranges from zero to one, where one indicates complete inequality. We obtained state-level GINI for 2006 directly from the American Community Survey [41].

#### Socio-economic deprivation

Because socio-economic deprivation can predict levels social trust [48], we operationalized this measure through a composite index derived from state-specific median household income, proportion of households in poverty, and proportion of households with less than a high school education, which are all validated area-based socio-economic indicators [49]. Following methods in our and others’ work [9,50], we used principal components analysis (PCA) with varimax rotation. PCA produced a one-factor solution explaining 78% of variance. We used the predicted standardized z-score variable.

#### Religious involvement

Religious involvement is distinct from social cohesion/capital [51], and at the state level can influence policies [52] thus could compete with social trust [53] to impact late HIV diagnosis. Data were from the 2007 U.S. Religious Landscape Survey, which is based on a representative sample of 35,556 adults living in the continental U.S. Further details on methodology are published [54]. We operationalized this measure using a composite index derived from the proportion of participants’ responses to doing the following activities at least once a week. The specific questions for the activities are “And still thinking about the church or house of worship where you attend religious services most often, please tell me how often, if ever, you do each of the following? (1) do community volunteer work through your place of worship, (2) work with children or youth at your place of worship, and (3) participate in social activities, such as meals, club meetings, or gatherings there.” We used PCA with varimax rotation, which yielded a one-factor solution that explained 76% of variance.

#### Health insurance

The Affordable Care Act (ACA) and health insurance coverage are structural factors that can influence HIV care continuum outcomes [55] aside from social trust. We also included the following *socio-demographic covariates* that may affect social trust as well the outcomes: population density, % of foreign born persons, % of population living in urban areas, % of population between the ages of 18 to 34 years old, and % of persons living in different house one year ago. We obtained the socio-demographic covariates and insurance status from the American Community Survey (ACS) 2013 (5-year estimates) [56]. Affordable Care Act (ACA) data came from Kaiser Family Foundation website [57]. States in 2009 that participated in Senate legislative hearings to enrol in ACA remained after the programme was signed into law in 2010.

All secondary data used for this study were aggregated at the state level, de-identified and not considered human subjects research. CDC surveillance data on HIV diagnoses, and BRFSS HIV testing data, Gallup social trust data, and socio-demographic covariates are all publicly available and provided online in accordance with each institution’s ethical and consent protocols.

### Statistical analysis

We examined significance of trends in late HIV diagnosis and mortality from 2009 to 2013 through regression analysis. We used correlation analysis to describe the relationship among the continuously distributed social trust and covariates.

For multivariable analyses, we used generalized structural equation modelling (GSEM) with robust standard errors clustered for *N* = 5 years. The family was negative binomial with log link to model the count of cases for the outcomes [58], and a Gaussian model for HIV testing. The count of late HIV diagnoses for each of the 5 years was modelled with state-specific total population as the offset variable in the regression. For all-cause mortality, the number of PLWHA in each year was the offset variable.

To examine racial/ethnic differences, we created an interaction term between race/ethnicity*social trust and entered it into the model, adjusting for covariates. We tested for statistically significant interactions with chi-square values. To examine the indirect effect of HIV testing on the late HIV diagnosis, we created an interaction term for race/ethnicity*HIV testing and entered it into the GSEM model with race/ethnicity*social trust.

We calculated the proportion of indirect to total effect (i.e., proportion mediated) and considered a 10% change meaningful mediation as a conservative estimate based on 15% proposed in indicator 9 of the National HIV/AIDS Strategy for reducing disparities in new diagnoses [59]. The indirect effect/total effect should be only computed when the total effect is not close to zero, specifically, ±0.20 because results may be unstable [60]. Mediating effects may be negative when the total effect is small due to suppression effects; this is known as inconsistent mediation [60]. We therefore only report proportion mediated for the interactions in the text, for those results consistent with mediation and describe those results where suppression effects occurred. The indirect effect is statistically significant if the confidence interval of the regression coefficient does not include zero [61]. Statistical significance for the indirect effect was examined via the bias corrected standard errors from bootstrapping with 500 repetitions and clustered for *N* = 5 years. All calculations were done directly in STATA 14.0 software [62].

## Results

**Figure 2.**
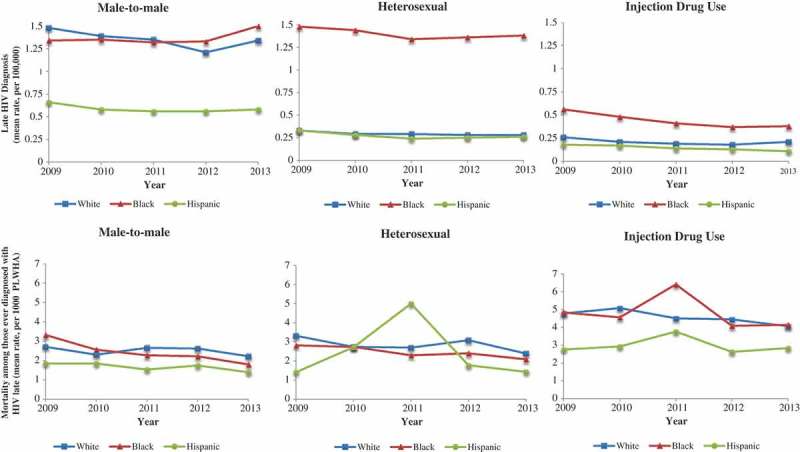
Five-year trends of late HIV diagnosis across race/ethnicity by transmission group (graphs on top) and all-cause mortality among people ever diagnosed with HIV late (bottom). We calculated rates based on data from 47 American states (Alaska and Hawaii, and New Hampshire were excluded due to missing or suppressed social trust or HIV/AIDS data; Washington D.C. for extremely high outlier values on HIV/AIDS data).

Trend analyses revealed a wide Black-White and Hispanic-White gap in late HIV diagnosis from 2009 through 2013 for heterosexual and IDU transmission groups. No trends in late HIV diagnosis across race/ethnicity were significant (i.e., all *p* > 0.05). Mortality rates appeared to decline for Blacks and Whites but remained stable for Hispanics in the MSM transmission group. Decreasing trends in mortality were only significant for Blacks in MSM (b = −0.33, se(0.10), *p* = 0.001, and heterosexual transmission groups (b = −0.18, se(0.08), *p* = 0.028 (Figure 2).

Social trust was negatively correlated with income inequality (*r* = −0.73, *p* < 0.01), % foreign born (*r* = −0.41, *p* < 0.01) and socio-economic deprivation (*r* = −0.52, *p* < 0.01), all factors hypothesized to erode social cohesion/capital [53]. Social trust and religious involvement was inversely related but not significant (*r* = −0.25, *p* = 0.08) (Table 1).

**Table 1.** Pearson product moment correlation coefficients among study variables

	1	2	3	4	5	6	7	8	9	10	11	12	13	14
1 Social trust	1.0													
2 Late HIV diagnosis	−0.72***	1.0												
3 All-cause mortality	−0.67***	0.95***	1.0											
4 Religious involvement^a^	−0.25	0.24	0.16	1.0										
5 Socio-economic deprivation^b^	−0.52***	0.17	0.06	0.51***	1.0									
6 Income inequality^c^	−0.73***	0.61***	0.65***	0.25	0.38**	1.0								
7 HIV testing^d^	−0.26	0.20	0.25	0.14	−0.13	0.19	1.0							
8 Urban/rural	−0.34*	0.39**	0.42**	0.03	−0.31*	0.24	0.47***	1.0						
9 Population density	−0.19	0.40**	0.53***	−0.25	−0.50***	0.28	0.38**	0.52***	1.0					
10 % Foreign born	−0.41**	0.44**	0.51***	−0.02	−0.28	0.38**	0.38**	0.80***	0.52***	1.0				
11 %Age18-34	−0.04	0.02	−0.05	0.39**	−0.04	−0.01	0.27	0.36*	−0.18	0.20	1.0			
12 Residential instability^e^	0.02	−0.25	−0.38**	0.28	0.29*	−0.28	−0.20	0.07	−0.60***	−0.05	0.43**	1.0		
13 Health insurance	0.50***	−0.27	−0.16	−0.58***	−0.71***	−0.27	0.02	−0.11	0.36*	−0.18	−0.22	−0.54***	1.0	
14 Affordable Care Act^f^	−0.04	0.01	0.06	−0.31*	−0.20	−0.02	0.18	0.30*	0.26	0.21	−0.13	−0.33*	0.25	1.0

*p < 0.05, **p < 0.01, ***p < 0.001 Analyses for late HIV diagnosis and all-cause mortality include 47 states (Alaska and Hawaii excluded due to missing independent variable data; New Hampshire excluded due to missing dependent data; Washington D.C. for extremely high outlier values). ^a^Composite variable indicating performing work or social activities at place of worship (higher is greater). ^b^Composite variable including median income, household poverty, and education level (higher is greater). ^c^Measured by the GINI coefficient. ^d^Age-adjusted HIV testing in past 12 months for Black, White and Hispanic only. ^e^Measured by % of persons who lived elsewhere in the previous year. ^f^Assessed with Spearman correlation coefficient because is a binary variable 1 = yes, 0 = no. All other coefficients are Pearson Correlations.

**Figure 3.**
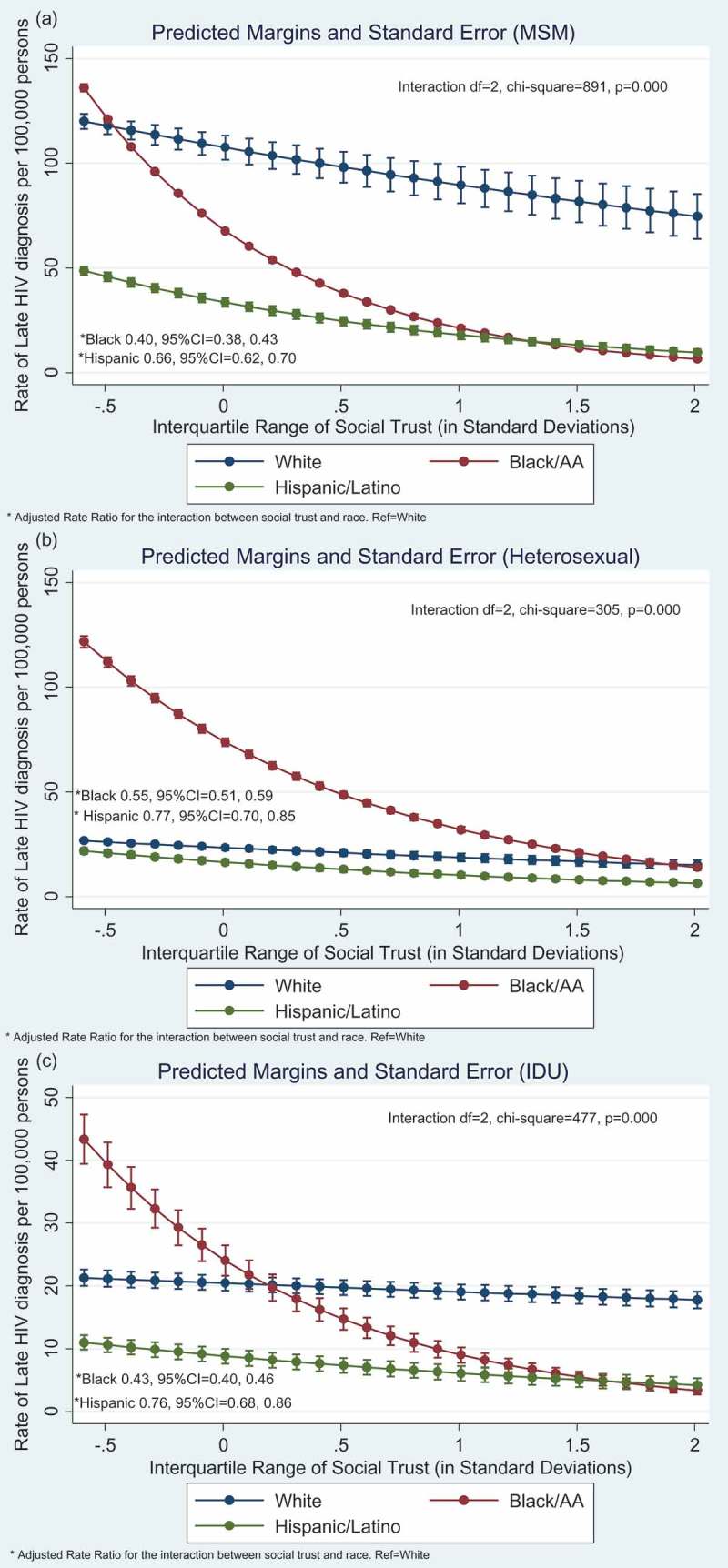
**Graphical display of racial/ethnic differences in the association between social trust and late HIV diagnosis. Results based on Model 2 (Supplement Table 1), which is adjusted for income inequality, socio-economic deprivation, religious involvement, % urban areas, population density, % foreign born, % age 18–34, residential instability, % insured, Affordable Care Act (yes/no). (a) male-to-male (MSM) transmission group, (b) heterosexual transmission group, and (c) injection drug use (IDU) transmission group**. ^a^Analyses conducted with Generalized Structural Equation Model (GSEM) using Negative binomial and log link, with robust standard errors clustered for *N* = 5 years of repeated data for *N* = 47 states, across *N* = 3 race groups.

Social trust was associated with lower rates of HIV diagnosis (Supplement Table 1, Model 1). Figure 3 displays racial/ethnic differences in the association between social trust predicting late HIV diagnosis, corresponding to the results in Supplement Table 1, Model 2. Racial/ethnic differences were significant across all transmission groups (i.e. test for interaction *p* < 0.001, Figure 3). The protective association was strongest for Blacks followed by Hispanics relative to Whites as seen by the sharp declining slope from left to right.

Supplement Table 1, Model 3 contains the results from the mediation analysis. HIV testing was significantly associated with higher late HIV diagnosis and that relationship varied by race/ethnicity across all transmission groups (i.e. test for interaction *p* < 0.001, results not displayed).

Compared to White MSM, HIV testing mediated 18% for Black MSM and −35% for Hispanic MSM. A positive indirect effect means the protective association between social capital and late HIV was strengthened whereas a negative percentage means the protective association was weakened such that HIV testing had a suppression effect [60]. Compared to White heterosexual, HIV testing mediated 26% for Black heterosexual. The indirect effect was not calculated for Hispanic heterosexual because the total effect was too small (i.e. less than +/−0.20) and the results could be unstable [60]. Compared to White IDU, HIV testing mediated 32% for Black IDU. The indirect effect for Hispanic IDU was not calculated because of a total effect near zero.

**Figure 4.**
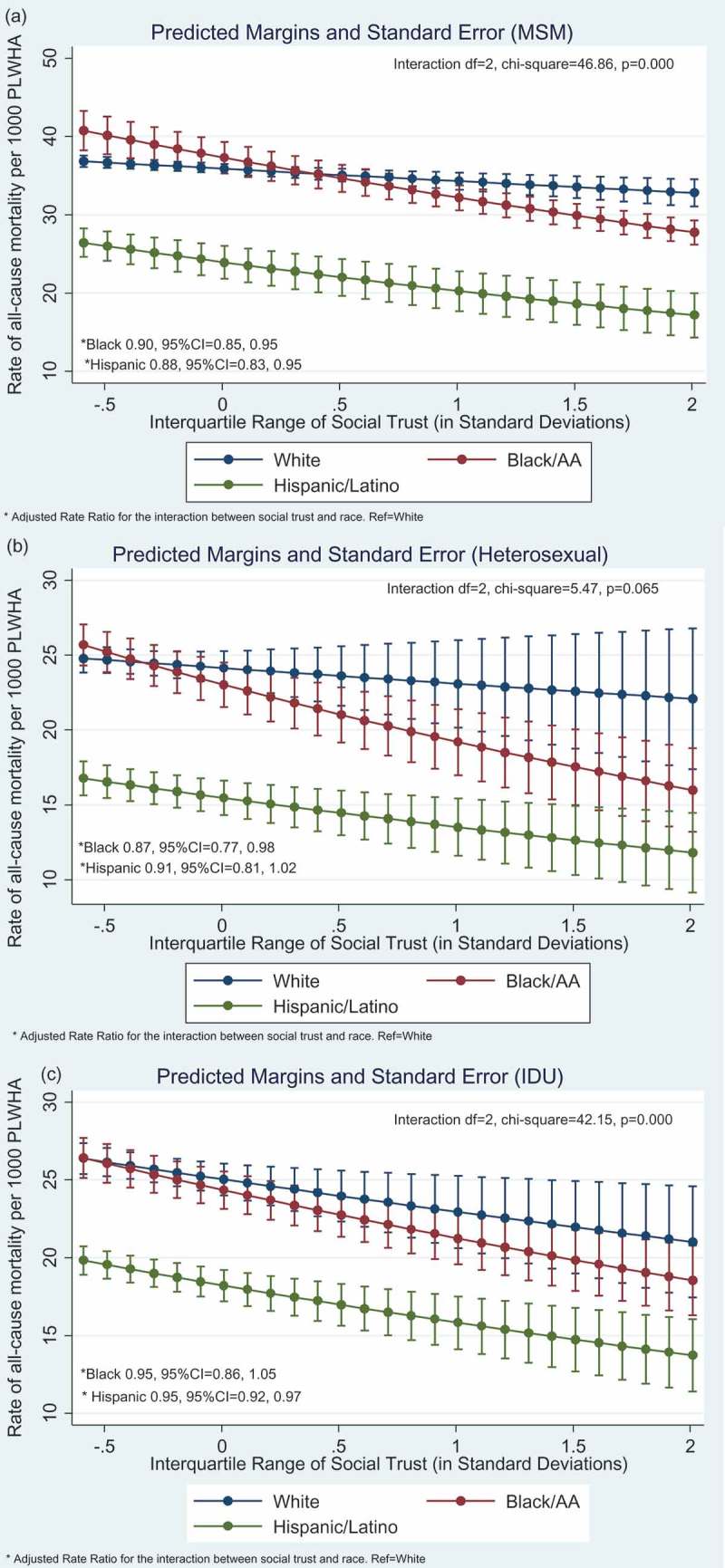
**Graphical display of race/ethnic differences in the association between social trust and all-cause mortality. Results based on Model 2 (Supplement Table 2), which is adjusted for income inequality, socio-economic deprivation, religious involvement, % urban areas, population density, % foreign born, % age 18–34, residential instability, % insured, Affordable Care Act (yes/no). (a) male-to-male (MSM) transmission group, (b) heterosexual transmission group, and (c) injection drug use (IDU) transmission** **group**. ^a^Analyses conducted with Generalized Structural Equation Model (GSEM) using negative binomial and log link, with robust standard errors clustered for *N* = 5 years of repeated data for *N* = 47 states, across *N* = 3 race groups. Except no deaths among Hispanic MSM in North Dakota in 2009, no deaths among Black IDU in Montana and Wyoming across all 5 years, and no deaths among Hispanic IDU in South Dakota for 2009 and 2010.

Social trust was associated with lower all-cause mortality (Supplement Table 2, Model 1). Figure 4 displays graphical plots of racial/ethnic differences in the association between social trust and all-cause mortality, corresponding to Supplement Table 2, Model 2. The protective association was stronger Blacks followed by Hispanics relative to Whites in the MSM transmission group. In the IDU transmission group, the protective association differed between Hispanics and Whites only.

**Figure 5.**
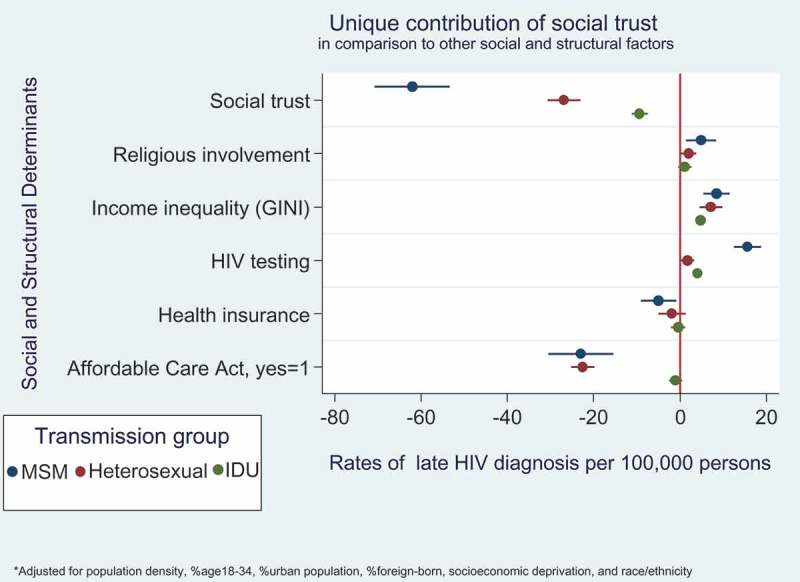
Descriptive plots showing the protective impact of social trust on late HIV diagnosis rates in comparison to competing social and structural factors. Separate models were conducted for each transmission group.

Figure 5 shows that higher levels of social trust was associated with 3-times lower late HIV diagnosis rates, especially in comparison to higher health insurance coverage and states with the Affordable Care Act (e.g. 60 vs. 21 per 100,000 persons, in the MSM group).

## Discussion

Given the persistent racial/ethnic disparities in HIV incidence and mortality in the US [3], we investigated racial/ethnic differences in the association between social trust – a potential protective social factor – and both primary and secondary HIV prevention outcomes [30], across 47 American states. Social trust had a robust protective association with late HIV diagnosis but marginal protective association with all-cause mortality. The direction of our findings is consistent with one other state-level ecological study that examined social capital and AIDS case rates [18] and a zipcode-level study that examined social capital and late HIV diagnosis in New York City [16].

The protective relationship between social trust and the HIV clinical outcomes was stronger for Blacks and Hispanics compared to Whites across the 3 major transmission groups. HIV testing mediated between 18% and 32% of racial/ethnic differences in the association between social trust and late HIV diagnosis, but for Blacks compared to Whites only. The per cent mediated is higher than the 15% reduction in disparities in new HIV diagnosis proposed in indicator 9 of the National HIV/AIDS Strategy [59].

These findings may inform actionable interventions given that Black MSM have the highest HIV incidence in the US and could benefit from investments in social factors that could attenuate disparities. Prior evidence indicates that social cohesion/capital is associated with greater individual likelihood and higher community-level rates of HIV testing [17,20,63]. Also, Black/African American MSM test for HIV at higher rates than White MSM [64]. The lack of differences for Hispanics needs further study. Perhaps for this group, issues related to heterogeneity among Hispanic/Latinos (e.g. Cubans vs. Mexicans), lower healthcare access, and acculturation may be more salient in reducing late HIV diagnosis [65].

Including social trust and other social cohesion/capital-related indicators in national surveys such as BRFSS and Current Population Survey (CPS) could facilitate novel ways to monitor determinants of HIV care continuum indicators at the state and local levels [66]. Our results add to a broader discussion of welfare politics and health and perhaps a prior debate about whether social cohesion/capital is less effective than macro-economic policies [67,68], but with a new focus on HIV/AIDS. For instance, one study found that non-progressive states with fewer safety net policies, the impact of higher social trust on lower smoking and days in poor health was stronger [44]. Government performance and innovation (e.g. structural balance between revenue and expenditures) was higher in states with higher social trust among individuals [69,70]. Specific to HIV/AIDS, states with higher spending on social services per person in poverty had significantly lower AIDS deaths rates [71]. The social trust associations in this study were robust net other competing economic and structural factors such as income inequality and ACA coverage. Thus, ours and other social cohesion/capital and HIV studies [18,34] encourage more systematic research on the topic.

There are some study limitations. Social trust is a valid but rough proxy for social cohesion and one indicator within collective efficacy [22]. However, social trust and other cohesion indicators are highly correlated (e.g. *r* > 0.80) and sometimes used as a composite index [18]. The social trust measure is an aggregate of responses within a state, which could mask heterogeneity across geography within the states. Race/ethnicity and socio-economic composition within neighbourhoods can predict social capital [48], including trust [72]. We tried to mitigate potential bias by controlling for urban-rural differences and population density but we could not account for sample bias nor adjust for socio-demographic factors of survey respondents because we did not have raw social trust data. Causal inference is also limited given that social trust could also be simultaneously affected by other exogenous factors and social processes occurring at the same time which in-turn influences the outcomes. Despite these limitations, we used several population based sources to research a topic with considerable impact for reducing racial/ethnic disparities in HIV care continuum outcomes.

This study should be replicated across other geographic levels and using other indicators of social cohesion/capital. Longitudinal studies in the US are also needed given evidence in international settings showing causal relations between social capital and reductions in HIV incidence [73].

## Conclusions

Social trust may promote earlier HIV testing, which can facilitate earlier HIV diagnosis, thus it can be a useful determinant to monitor the relationship with HIV care continuum outcomes especially for racial/ethnic minority groups disproportionately infected by HIV.
